# Exhaustive Training Increases Uncoupling Protein 2 Expression and Decreases Bcl-2/Bax Ratio in Rat Skeletal Muscle

**DOI:** 10.1155/2013/780719

**Published:** 2013-01-09

**Authors:** W. Y. Liu, W. He, H. Li

**Affiliations:** ^1^Department of Sports Medicine, Shanghai University of Sport, Shanghai 200438, China; ^2^Department of Rehabilitation, Shanghai Jing'an Geriatric Hospital, Shanghai 200040, China; ^3^Department of Physical Education, Neijiang Normal University, Sichuan 641100, China

## Abstract

This work investigates the effects of oxidative stress due to exhaustive training on uncoupling protein 2 (UCP2) and Bcl-2/Bax in rat skeletal muscles. A total of 18 Sprague-Dawley female rats were randomly divided into three groups: the control group (CON), the trained control group (TC), and the exhaustive trained group (ET). Malondialdehyde (MDA), superoxide dismutase (SOD), xanthine oxidase (XOD), ATPase, UCP2, and Bcl-2/Bax ratio in red gastrocnemius muscles were measured. Exhaustive training induced ROS increase in red gastrocnemius muscles, which led to a decrease in the cell antiapoptotic ability (Bcl-2/Bax ratio). An increase in UCP2 expression can reduce ROS production and affect mitochondrial energy production. Thus, oxidative stress plays a significant role in overtraining.

## 1. Introduction

The mechanism ofovertraining (OT) remains poorly understood.One common theory considered is glycogendepletion[1].Ourprevious study showed that glycogen was not depleted in rat skeletal muscles after continuous exhaustive training, which does not support theglycogendepletionhypothesis [2].Overtraining can be caused byreducedmuscle mitochondrialfunction,reducing glycogen breakdown and decreasing energy production.ExcessiveROS can influence the reduction of mitochondrialfunction due to continuousexhaustive training. ROS can be associated with overtraining, inducing the opening of the mitochondrial permeability transition pore (MPTP) [3]. Low molecular weight molecules (<1.5 kDa) equilibrate across the inner membrane when the MPTP opens, causing mitochondrial swelling and outer membrane rupture. The opening of the MPTP is considered the “point of no return,” after which the myocyte is irreversibly committed to necrotic or apoptotic death pathways [4].

Many pathways can lead to cell apoptosis. One of the mitochondrial-mediated pathways, including the Bcl-2 family, is best characterized and considered critical in regulating apoptosis. In the Bcl-2 family, Bax protein is mainly located in the cytoplasm, which migrates to the outer mitochondrial membrane, forms dimer and oligomer under the apoptosis signal stimulation and combines with the adenine nucleotide translocator of the MPTP complex or voltage-dependant anion channel on the outer mitochondrial membrane. This combination occurs either directly or through the Ca^2+^ released from the endoplasmic reticulum-induced MPTP opening, leading to apoptosis [5]. The main protein inhibiting apoptosis, Bcl-2, anchors to the mitochondria, endoplasmic reticulum, and nuclear envelope of the cytoplasmic side. This action maintains mitochondrial membrane integrity through competitive inhibition of Bax mediated by mitochondrial membrane protein channel formation [6], controlling the opening of PMTP, inhibiting Ca^2+^ transmembrane flow, inhibiting caspase-3 activation, and preventing apoptosis. Apoptosis caused by continuous exhaustive training can result from ROS-induced permeability transition pore opening [7]. A study by Kim et al. on endoplasmic reticulum stress [8] states that Bax inhibitors can reduce ROS accumulation by regulating cytochrome P450 2E1. This suggests that ROS and Bax are closely associated.

UCP2 can regulate ROS generation. Echtay observed that mild uncoupling reduces the mitochondrial production of ROS [9]. ROS are important mediators of tissue damage. A recent study also showed that UCP2 influences apoptosisregulation in different cell systems [10]. The present study investigates the effects of oxidative stress due to exhaustive training on UCP2 and Bcl-2/Bax in rat skeletal muscles. Particularly, this study aims to evaluate the effects of oxidative stress on tissue damage and determine the relationship between oxidative stress and overtraining.

## 2. Materials and Methods

### 2.1. Animals

Eighteen 8-week-old female Sprague-Dawley (SD) rats from Shanghai Sino-British Sipper/BK Lab Animal, Ltd. were used. The animals were housed at 25°C with an inverted 12 h light-dark cycle and fed ad libitum. All experiments were approved by the Ethics Committee of Shanghai University of Sport and complied with the National Regulation for Administration of Laboratory Animals. Prior to training, all rats were adapted to treadmill running for one week. The adaptation phase consisted of treadmill running 6 days/week for 5 min at a speed of 10 m/min. At the end of this period, the rats were randomly divided into three groups: the control group (CON), the trained control group (TC), and the exhaustive trained group (ET). Six rats were housed per cage, with the trained animals stored in cages separate from those of untrained animals but in the same room of the animal housing facility.

### 2.2. Training Protocol

The training protocol was designed to induce a training-to-OT continuum (Table 1) [11]. Both the training volume and intensity were gradually increased in the first six weeks. During the last three weeks, the TC and ET groups were maintained at the same exercise intensity (the same speed and grade); however, the ET group was trained longer until exhaustion. The exhaustion was defined as the point at which the animals failed to get off the shock grid and thus had to be manually returned to the front of the treadmill for three consecutive occasions [12]. The actual training duration of the ET group ranged from 180 min to 200 min in the seventh week and 60 min to 80 min in the eighth and ninth weeks. The training week consisted of six consecutive days of training sessions followed by one rest day. A motorized treadmill with adjustable inclination was used (DSPT202, Qianjiang Technology Company, Hangzhou, China).

### 2.3. Tissue and Blood Sample Collection

The TC and the ET groups were sacrificed after 36 h after the last training session to avoid acute exercise effects. The control group was sacrificed at the end of the nine weeks. All rats were anesthetized with pentobarbital (40 mg/kg body weight). Blood was rapidly collected from the abdominal aorta; plasma was separated by centrifugation and then stored at −78°C for further analysis [12]. The samples were dissected from the darker side of the red gastrocnemius muscle. Half of the samples were formalin-fixed, whereas the remaining samples were frozen in liquid nitrogen and stored at −78°C.

### 2.4. Hemoglobin (Hb) Assay

Hb was analyzed by an automatic cytometer (BC-3000, Shenzhen Mindray Bio-Medical Electronics Co., Ltd., Shenzhen, China).

### 2.5. Mitochondrial Isolation from Skeletal Muscles

The red gastrocnemius muscles were freed of excess fat and connective tissue, finely minced, and washed in a medium containing 20 mM HEPES, 2 mM MgCl_2_, 120 mM KCl, 5 g/L BSA, with pH of 7.4. The tissue fragments obtained were homogenized with the above medium (1 : 8, w/v). The homogenate was then centrifuged at 600 g for 10 min twice. The resulting supernate was centrifuged at 17000 g for 10 min. The pellet was homogenized with the above medium (1 : 10, w/v) and then centrifuged again at 7000 g for 10 min. The pellet was washed and resuspended in a suspension medium (300 mM sucrose, 0.1 mM EGTA, 2 mM HEPES, pH 7.5) and centrifuged at 3500 g for 10 min. The pellet was resuspended in the suspension medium described above to determine SOD, XOD, and ATPase activities and MDA [13]. The pellet was broken down by sonication (JY92 Ultrasonic Cell Pulverizer, Ningbo Scientz Biotechnology Co., Ltd.) before analysis.

### 2.6. Protein Concentration

Protein concentrations were determined by Bio-Rad Bradford protein assay.

### 2.7. Mitochondria Oxidative Stress Markers and ATPase

Mitochondria SOD, XOD, ATPase activities, and MDA concentrations were measured using a commercial kit (Nanjing Jiancheng Bioengineering Institute, China).

### 2.8. Skeletal Muscle UCP2 Western Blot Analysis

After the red gastrocnemius muscles were homogenized in an ice-cold lysis buffer,whole-tissues homogenates were centrifuged at 14,000 g for 20 min,and the supernates were collected. After quantitation of proteinconcentration, equal amounts of proteins (30 *μ*g) were electrophoresed on sodium dodecyl sulfate polyacrylamide gel and then transferred onto polyvinyl difluoride. Themembranes were blocked with 5% nonfat dry milk in Tris-buffered saline for 1 h at roomtemperature, and incubated overnight at 4°C with primary antibodies (Wuhan Boster Bio-Engineering Ltd. Co., China).The membranes were then incubated with appropriate horseradish peroxidase-linked secondary antibodies (Pierce, Rockford,IL, USA) for 2 h at room temperature and visualized using an enhanced chemiluminescence detection system (Pierce, Rockford, IL, USA) [14]. Following western blot development, the relative abundance of UCP2 was determined by densitometry. The band intensities of the exposed film were analyzed using Image J software. 

### 2.9. Immunohistochemical Staining

Bcl-2 and Bax from the red gastrocnemius muscle of each group were determined by the streptavidin-biotin complex (SABC) method. The paraffin sections (4 *μ*m) from each group were dehydrated in xylene and graded ethanol series and added in order with the primary antibody (rabbit polyclonal antibody, Santa Cruz, USA), biotinylated secondary antibody (goat serum 1 : 100, Wuhan Boster Co., China), SABC reagents, and diaminobenzidine solution (Wuhan Boster Co., China). For negative control, the sections were treated with PBS instead of primary antibody. Ten sample sections from each group were selected for analysis. More than five visual fields were observed per section. The following equation was used: the positive percentage of each protein = protein − positive cells/all cells × 100%.

### 2.10. Statistical Analysis

 Statistical analyses were performed using SPSS 13.0 for Windows (SPSS Inc., Chicago, IL, USA). The results are presented as means ± standard deviation (SD). We used one-way ANOVA followed by least significance difference post hoc test to compare the means of the three groups. If data failed the normality test, the Kruskal-Wallis one-way ANOVA on ranks and Tukey's post hoc test were used. *P* < 0.05 was considered statistically significant.

## 3. Results

### 3.1. Body Weight

Both trained groups exhibited a significant decrease in body weight relative to the CON group at the end of the ninth week of training (*P* < 0.05), but no significant difference was indicated between the TC and the ET groups (Table 2).

### 3.2. Hb, Concentration of MDA, and Activities of XOD, SOD, and ATPase

Hb was significantly decreased in the ET group compared with the CON and the TC groups (*P* < 0.001) (Table 3).MDA concentration was significantly increased in the ET group compared with the CON group (*P* < 0.05). SOD activity was significantly decreased in the ET group compared with the CON group (*P* < 0.05). The SOD/MDA ratio was significantly increased in the TC group compared with the CON group (*P* < 0.05), but the SOD/MDA ratio was significantly decreased in the ET group than that in the CON group (*P* < 0.001). XOD activity was significantly increased in the ET group compared with the CON group (*P* < 0.05). Meanwhile, ATPase activity was significantly decreased in the ET group compared with the CON group (*P* < 0.001).

### 3.3. Muscle UCP2 Protein Expression

**Figure 1:**
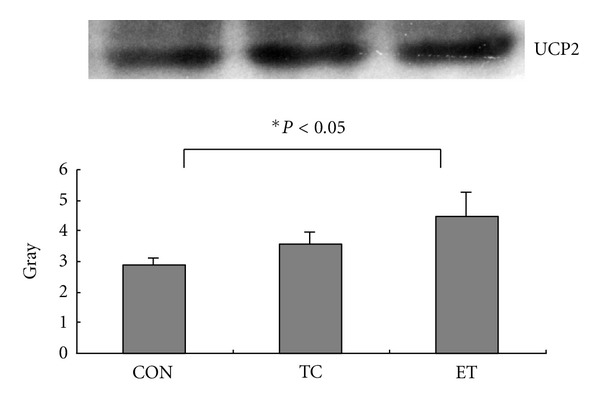
UCP2 protein expression in rat gastrocnemiusmuscle.

 The skeletal muscle UCP2 skeletal muscle UCP2 expression was significantly higher in the ET group than that in the CON group (*P* < 0.05). However, no difference in the UCP2 expression was indicated between the TC and the CON groups (Figure 1).

### 3.4. Bax and Bcl-2 Protein Expression

**Figure 2:**
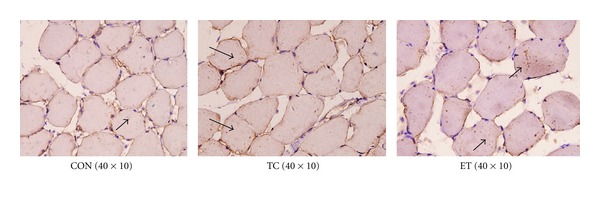
Positive immunoreactivity of Bax in each group.

**Figure 3:**
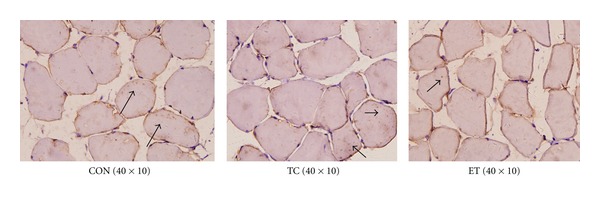
Positive immunoreactivity of Bcl-2 in each group.

Bax protein expression was higher in the ET group than in the CON group (Figure 2). Bcl-2 protein expression was lower in the ET group than in the CON group (Figure 3). The Bcl-2/Bax ratio was significantly (*P* < 0.01) decreased in the ET group compared with the CON group (Table 4). 

## 4. Discussion

In the present study, continuous exhaustive training was shown to cause skeletal muscle mitochondrial MDA concentration, and XOD activity significantly increased. However, SOD activity in the rats was suppressed. Continuous exhaustive training damaged the balance between the intracellular oxidative and antioxidant factors. The rats simultaneously showed a rapid decrease in motion ability, standing dull hair, decreased locomotor activity, lack of response, and decreased Hb, leading to the OT state [15, 16]. This study found that the Bax expression of promoted apoptosis increased and Bcl-2 expression inhibited apoptosis decreased after continuous exhaustive training. The significant decrease in the ratio of Bcl-2/Bax showed that continuous exhaustive training promoted the apoptosis process. Currently UCP2 is known to regulate ROS concentration and apoptosis. After continuous exhaustive training in our experiments, skeletal muscle mitochondria UCP2 expression increased significantly, which can be related to the increase in the induction of MAD concentration and the reduction in the ratio of Bcl-2/Bax [17].

### 4.1. Oxidant and Antioxidant

ROS can be produced from numerous sources during exercise. These sources include the mitochondrial electron transport chain and xanthine oxidase system, among others [18]. Our study indicated that after OT mitochondrial MDA concentration and XOD activity increased in rat red gastrocnemius muscles. MDA, which indirectly reflects the degree of ROS on membrane lipid peroxidation, is one of the products of membrane lipid peroxidation. XOD is the main enzyme of the xanthine oxidase pathway. This enzyme is involved in the pathophysiology of ischemia-reperfusion syndrome and can lead to tissue damage after an exhaustive bout of exercise [19], Thus, the increases in XOD activity and MDA concentration indicated that continuous exhaustive training caused by overtraining enhanced tissue damage. On the other hand, SOD activity decreased, suggesting that OT increases ROS generation and inhibits the scavenging ability of ROS [20]. These changes can be related to allosteric downregulation of the enzymes and enzyme inactivation due to overwhelming oxidative stress [21]. Studies suggest thatincreased oxidative stress influences the pathophysiology of overtraining. The weakened responses of oxidative stress and antioxidant capacity to exercise in the overtraining state can be associated with the inability to exercise effectively and impaired adaptation to exercise [22].

Continuous exhaustive training can significantly affect ROS accumulation. The mitochondrial electron transport chain (ETC) of oxidative phosphorylation is identified as a major site for cellular ROS generation.As electrons pass through the complexes of the ETC, some of these electrons leak to molecular oxygen, thus forming superoxides [23]. When energy consumption in the tissue increased sharply during the exhaustive training, mitochondrial oxygen consumption also increased, and ROS generation rose. Lactic acid concentration increases caused by exhaustive training induce a synergistic effect on ROS production [24].

Mitochondrial ROS production highly depends on the membrane potential generated by the proton gradient formed across the inner mitochondrial membrane [25]. High membrane potential is shown to stimulate ROS production. One view states that in the presence of a large mitochondrial matrix such as in stress situations, O_2_^•−^  can be activated in the endometrial stromal side of uncoupling proteins (UCPs). This activation leads to proton transfer and mitochondrial membrane solution coupling, thus reducing ROS generation [26, 27]. ROS production and mitochondrial proton leak are mediated by UCP2.According to feedback loop theory [28], activation of proton leak decreases the mitochondrial membrane potential, thus limiting mitochondrial ROS generation. This effect shows that the UCP2 can reduce ROS generation in oxidative stress and protect the cell from ROS-induced damage. Thus, UCP2 can regulate the concentration of intracellular ROS.Exercise as a stress factor can induce a UCP2 response. Studies showed that a one-time exercise temporarily increases the impression of UCP2b mRNA; endurance exercise shows no such effect [29]. In the present study, UCP2 significantly increased in the ET group after exhaustive training but not in the TC group, which indicates inflammation [30]. Continuous exhaustive training stimulates ROS generation, which results in increased expression of mitochondrial UCP2 protein to protect cells from damage caused by inflammation. Thus, UCP2 is considered an important number in the antioxidant system. The transcription of the UCP2 gene itself is highly inducible under the conditions of oxidative stress [17, 27]. 

### 4.2. Uncoupling and Energy Loss

UCP2 can affect mitochondrial energy production when ROS generation is inhibited. Studies found that the ATP content of UCP2-overexpressing islets was reduced by 50% [31] and that ATP stores are reduced by 15% to 30% in UCP2-overexpressing hepatocytes [32, 33]. These findings suggest that increasing mitochondrial proton leak induces a decrease in ATP synthesis and reduces the efficiency of energy metabolism. Bouillaud proposed a metabolic hypothesis in which UCP2 acts through a transport distinct from the proton transport. He asserted that this transport activity decreases the mitochondrial oxidation of glucose-derived pyruvate [34]. These actions increase the influence of UCP2 on cellular metabolism.In the present study, UCP2 expression was significantly increased in the ET group; however, mitochondrial ATP synthase activity was significantly reduced. Thus, OT induced by exhaustive training can decrease ATP synthesis and increase ROS production, enhancing UCP2 expression. UCP2 overexpression mediates ROS production and induces an imbalance between energy metabolism and ROS elimination, which reduces the efficiency of mitochondrial energy metabolism. Mild uncoupling can diminish mitochondrial superoxide production, increasing protection against diseases and tissue damage by a small energy loss [28].However, a substantial energy loss can occur after continuous exhaustive training. This loss can affect exhaustive training-induced OT. Westerblad and Allen [35] indicate that prolonged increases in ROS are likely to induce posttranslational changes in various proteins, which can directly affect contractile function. Decline in exercise ability may be closely associated with exercise-induced ROS-mediated changes in the ryanodine receptor (RyR1) can cause continuing decreases in sarcoplasmic reticulum (SR) Ca^2+^ release [36].

### 4.3. UCP2 and Apoptosis

UCP2 does not only suppress mitochondrial ROS; it also regulates apoptosis in various cell systems [22]. Cultured adult rat cardiomyocytes exposed to free fatty acids were shown to exhibit a dose-dependent increase in apoptosis and a significant increase in UCP2 expression. RNA interference and UCP2 knockdown reduced free fatty acid-induced apoptosis in cardiomyocytes [37]. This finding suggests that an increase in UCP2 expression results in increased apoptosis. A study on A549 cells under hypoxic conditions indicated [38] that UCP2 showed antiapoptotic properties. UCP2 overexpression inhibited ROS accumulation and apoptosis, as well as the release of cytochrome c, and reduced the activation of caspase-9. The aforementioned studies indicate that UCP2 has a regulating effect on ROS and cell apoptosis.

Apoptosis isa complexprocess involving several cellular proteins. A study suggested that Bcl-2/Baxexpressionbalance determinessurvival or death following apoptosis [39]. We found that OT led to the elevation of Bax expression and decrease in Bcl-2 expression in muscle tissues. TheBcl-2/Baxratio was significantlyreduced, suggesting that continuousexhaustive training-inducedROS resultingfrom overtrainingcoulddamagecells,leaving the red gastrocnemiusmusclewith decreasedantiapoptoticability. ROS can activate the early apoptotic signaling pathway MAPK (such as SAPK/JNK, ERK1/2, and p38) [40]. In such a process, ROS can inhibit Bcl-2 expression and promote cell apoptosis. Studies showed that by maintaining mitochondrial membrane integrity, Bcl-2 increases the outflow of protons in the mitochondria and inhibits the decrease in membrane potential. Bax may bind to voltage-dependant anion channel and open a permeability transition pore, inducing a decrease in membrane potential and promoting cell apoptosis [41–43]. Continuousexhaustive training alters the Bcl-2/Bax balance, thus increasingthe likelihood ofapoptosis.

In summary, exhaustive training increases the ROS in red gastrocnemius muscles, decreases SOD activity, and causes oxidative stress.Extensive ROS production increases UCP2 expression, which modulates the membrane potential and decreases ROS production. However, the increase in UCP2 expression also leads to a decline in mitochondrial energy metabolism.These effects can significantly affect exhaustive training-induced OT. On the other hand, ROS accumulation can increase Bax expression and reduce Bcl-2 expression, promoting cell apoptosis.These behaviors can be related to a pathological change after OT.

### 4.4. Perspective

The currentproblem is thatunder normal circumstances,the ATP/ADP ratioregulates the cytochromec oxidase (CcO)activity [44]. CcO is the terminal and rate-limiting enzyme of the respiratory chain.When theATP/ADP ratioincreases, ATPallosterically inhibits the CcO enzyme [45]. After continuousexhaustive training,the decreased ATP synthesis inmitochondria results inreducedfeedbackinhibition of CcO activity, which needs further investigation.CcOinhibitionknown as the feedbackto maintaina lowmitochondrial membranepotentialΔΨmand lowerROS levels. Thus, ifCcO activityincreases, afurther increase in ROSlevels is promoted, creating the viciouscirclephenomenon.

## Figures and Tables

**Table 1:** Training protocol.

Weeks	TC group			ET group		
	Speed (m/min)	Grade (%)	Time (min)	Speed (m/min)	Grade (%)	Time (min)
1	15	2	40	15	2	40
2	20	10	50	20	10	60
3	25	10	60	25	10	90
4	30	5	60	30	5	120
5	30	5	60	30	5	120
6	30	8	60	30	8	120
7	35	10	30	35	10	Exhaustion*
8	35	15	30	35	15	Exhaustion*
9	35	15	30	35	15	Exhaustion*

*Exhaustion was defined as the point at which the animals failed to get off the shock grid and had to be manually put back to the front of the treadmill for three consecutive occasions.

**Table 2:** Body weight change during the training (g).

Week	*n*	CON	TC	ET
0	6	225.75 ± 5.66	224.40 ± 4.24	228.68 ± 7.39
6	6	284.62 ± 18.19	270.95 ± 18.58	283.17 ± 15.68
9	6	296.75 ± 19.31	266.63 ± 16.41*	268.28 ± 19.53*

*Compared with CON, *P < 0.05. Means ± SD.

**Table 3:** Effect of training on Hb and mitochondrial parameters.

	CON (*n* = 6)	TC (*n* = 6)	ET (*n* = 6)	*P* value
Hb, g/L	121.9 ± 5.9	119.7 ± 4.5	99.2 ± 8.6^aa,bb^	< 0.01
SOD, U/mgprot	19.04 ± 2.42	21.12 ± 4.40	15.17 ± 5.56^b^	< 0.05
MDA, nmol/mgprot	15.52 ± 1.93	14.78 ± 0.82	18.40 ± 3.54^a,b^	< 0.05
SOD/MDA	1.23 ± 0.12	1.42 ± 0.24^a^	0.80 ± 0.20^aa,bb^	< 0.05
				< 0.01
XOD, U/mgprot	5.79 ± 0.72	7.49 ± 0.81	8.18 ± 1.20^a^	< 0.05
ATPase, U/mgprot	12.80 ± 0.45	10.61 ± 0.70	6.95 ± 0.17^aa^	< 0.01

^a,aa^Compared with CON: ^a^ *P *< 0.05, ^aa^ *P *< 0.01; ^b,bb^compared with TC: ^b^ *P *< 0.05, ^bb^ *P *< 0.01. Means ± SD.

**Table 4:** Changes in Bcl-2 and Bax protein expression in rat skeletal muscle.

Group	*n*	Bcl-2 positive rate (%)	Bax positive rate (%)	Bcl-2/Bax ratio
Con	6	19.29 ± 6.95	12.56 ± 2.99	1.49 ± 0.30
TC	6	17.67 ± 8.23	23.26 ± 6.05*	0.76 ± 0.32*
ET	6	12.22 ± 5.24	28.85 ± 7.75**	0.46 ± 0.23**

^∗,∗∗^Compared with CON: *P < 0.05, **P < 0.001. Means ± SD.
